# Small-Molecule Inhibitors Targeting Proteasome-Associated Deubiquitinases

**DOI:** 10.3390/ijms22126213

**Published:** 2021-06-09

**Authors:** Seonghyeon Moon, Srinivasan Muniyappan, Sung-Bae Lee, Byung-Hoon Lee

**Affiliations:** 1 Department of New Biology, Daegu Gyeongbuk Institute of Science and Technology (DGIST), Daegu 42988, Korea; t7t7t4t8@dgist.ac.kr (S.M.); srinivasan@dgist.ac.kr (S.M.); 2 Protein Dynamics-Based Proteotoxicity Control Lab, Basic Research Lab, Daegu Gyeongbuk Institute of Science and Technology (DGIST), Daegu 42988, Korea; sblee@dgist.ac.kr; 3 Center for Cell Fate Reprogramming & Control, Daegu Gyeongbuk Institute of Science and Technology (DGIST), Daegu 42988, Korea; 4 Department of Brain & Cognitive Sciences, Daegu Gyeongbuk Institute of Science and Technology (DGIST), Daegu 42988, Korea

**Keywords:** proteasome, proteolysis, deubiquitinase, USP14, UCH37, RPN11, small-molecule inhibitors, IU1, capzimin

## Abstract

The 26S proteasome is the principal protease for regulated intracellular proteolysis. This multi-subunit complex is also pivotal for clearance of harmful proteins that are produced throughout the lifetime of eukaryotes. Recent structural and kinetic studies have revealed a multitude of conformational states of the proteasome in substrate-free and substrate-engaged forms. These conformational transitions demonstrate that proteasome is a highly dynamic machinery during substrate processing that can be also controlled by a number of proteasome-associated factors. Essentially, three distinct family of deubiquitinases–USP14, RPN11, and UCH37–are associated with the 19S regulatory particle of human proteasome. USP14 and UCH37 are capable of editing ubiquitin conjugates during the process of their dynamic engagement into the proteasome prior to the catalytic commitment. In contrast, RPN11-mediated deubiquitination is directly coupled to substrate degradation by sensing the proteasome’s conformational switch into the commitment steps. Therefore, proteasome-bound deubiquitinases are likely to tailor the degradation events in accordance with substrate processing steps and for dynamic proteolysis outcomes. Recent chemical screening efforts have yielded highly selective small-molecule inhibitors for targeting proteasomal deubiquitinases, such as USP14 and RPN11. USP14 inhibitors, IU1 and its progeny, were found to promote the degradation of a subset of substrates probably by overriding USP14-imposed checkpoint on the proteasome. On the other hand, capzimin, a RPN11 inhibitor, stabilized the proteasome substrates and showed the anti-proliferative effects on cancer cells. It is highly conceivable that these specific inhibitors will aid to dissect the role of each deubiquitinase on the proteasome. Moreover, customized targeting of proteasome-associated deubiquitinases may also provide versatile therapeutic strategies for induced or repressed protein degradation depending on proteolytic demand and cellular context.

## 1. Introduction

**Figure 1:**
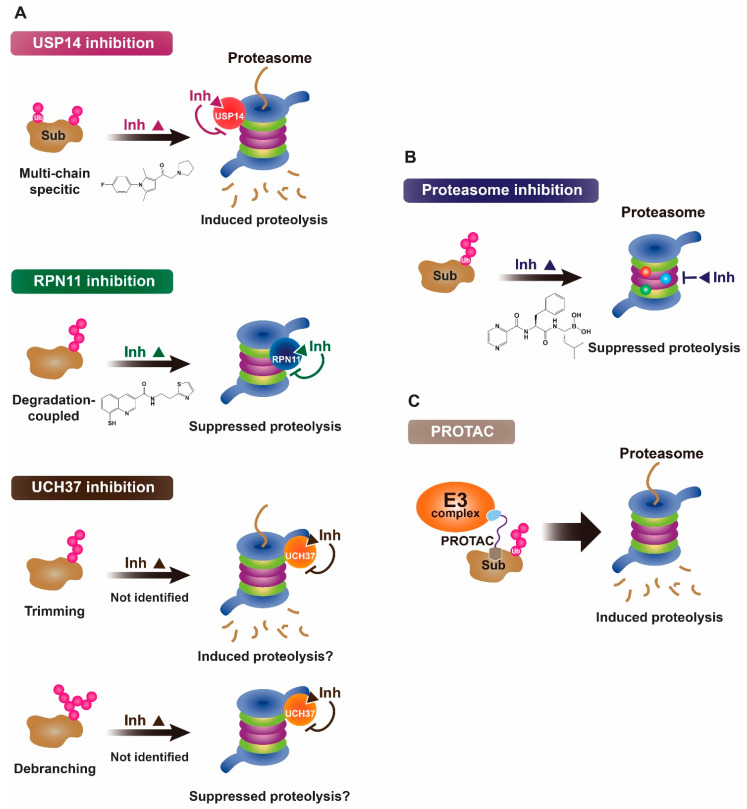
Proposed working mechanisms of proteasomal deubiquitinase inhibitors and their comparison to proteasome inhibitor and PROTAC. (**A**) (Top) USP14’s multi-chain specific cleavage activity can be selectively targeted by USP14 inhibitors (e.g., IU1 is shown as an example), resulting in induced degradation of substrates. (Middle) Degradation-coupled RPN11 activity can be selectively inhibited such as by capzimin as shown. RPN11 inhibition can strongly suppress the proteasome-mediated substrate degradation. (Bottom) UCH37 specific inhibitors–which have not been developed yet–may exert differential effects on proteolysis depending on the type of ubiquitin conjugates. Unbranched or poorly ubiquitinated substrates might be highly subject to UCH37’s trimming activity, and its specific inhibition may lead to induced protein degradation. By contrast, degradation of branched ubiquitin conjugates is likely to be attenuated by UCH37 inhibition. (**B**,**C**) Proteasome inhibitor (e.g., bortezomib as shown) and PROTAC are depicted as examples of proteolysis suppressor and inducer, respectively. Color-coded circles in proteasome at B indicate each pair of proteasome’s active sites. PROTAC is a chimeric compound closely linking E3 and target substrate, thus facilitating the ubiquitination process. Inh, inhibitor. See the text for more details.

The ubiquitin-proteasome system (UPS) represents a crucial cellular mechanism for highly regulated proteolysis and protein quality control process in eukaryotes [1,2]. The 26S proteasome is a large multi-subunit protease of ~2.5 MDa for selective degradation of intracellular proteins that are tagged by ubiquitins [3,4]. Recent findings indicate that proteasome is actively adapted to a large network of protein interactions for discrete degradation events, and such adaptability may also be controlled through a multitude of proteasome’s conformational transitions [5,6,7,8,9]. Notably, deubiquitinases (DUBs), which exclusively reverse the ubiquitination process in the UPS, are also critically associated with the proteasome [10,11,12]. In mammals, the regulatory particle (RP) of the 26S proteasome contains three major classes of DUBs–USP14 (Ubp6 in budding yeast), RPN11 (also known as PSMD14), and UCH37 (also known as UCH-L5) (Figure 1) [3,11,12,13]. USP14/Ubp6 is a reversible interactor with the proteasome, and its activity can be highly enhanced by association with the proteasome [12,14,15,16]. USP14 is capable of sparing the substrates from degradation prior to the proteasome’s commitment step and shows remarkable preference for multi-chain bearing ubiquitin conjugates [16,17,18]. By contrast, RPN11 is an integral subunit of the proteasome, and this metalloprotease is essentially coupled to substrate degradation in an ATP-dependent manner [11,19,20]. Although USP14 and RPN11 may mediate opposite proteolytic consequences, both of the enzymes apparently share a similar en bloc or proximal cleavage mechanism [11,17]. The function of UCH37 on the proteasome remains to be further established because this DUB may distally trim the ubiquitin chains for rescuing the substrates from degradation but also can selectively debranch the K48-linkage among a complex mixture of bifurcate ubiquitin conjugates for enhanced substrate degradation [12,21,22]. DUBs are emerging as attractive therapeutic targets because they may control the turnover rate of a number of intracellular proteins, including ones that might be highly deregulated in the disease states [23,24]. The isopeptidase activities of DUBs can be selectively inhibited by catalytic site-directed drug-like compounds. Moreover, recent advances in developing robust screening technologies with more refined chemical libraries have successfully yielded promising small-molecule DUB antagonists of active site-directed inhibitors as well as allosteric inhibitors [24,25,26,27]. Specific DUB inhibition on the proteasome is particularly appealing because each proteasome-associated DUB can exert distinct influence over the proteolytic outputs (Figure 1A). Therefore, it is not surprising that considerable efforts from academia and industry have also been put towards developing drug-like molecules for targeting proteasome-associated DUB activities [23,24,28]. Such specific DUB inhibitors at the proteasome not only offer exciting degradation-based therapeutic strategies but also serve as valuable chemical tools to reveal novel deubiquitination biology for dynamic proteasome function. In this article, we highlight recent progress in developing DUB inhibitors for specifically targeting proteasome-associated deubiquitinases, and their potential application in human diseases.

## 2. Proteasomal Deubiquitinases as Therapeutic Targets

For the past decades, the UPS has been clearly recognized among the most important drug targets because of its critical contribution to protein homeostasis, signaling pathways, and cellular physiology; its deregulation or genetic alteration is intimately associated with human pathogenesis [29,30,31,32,33]. The success story of proteasome inhibitors for cancer therapy highlights the clinical importance of the UPS as valid targets that can be even further expanded into various aspects of the proteolytic system and other types of pathophysiology [34,35]. In fact, a recently emerging novel paradigm of “induced proteolysis”, such as by PROteolysis TArgeting Chimera (PROTAC), can chemically harness the endogenous ubiquitination machinery for targeted protein degradation (Figure 1B) [36,37].

As opposed to proteasome inhibition, this new concept defines the UPS as yet another class of extraordinary drug target for effectively disposing of the conventionally intractable or “undruggable” disease-associated proteins (Figure 1) [24,38]. In the similar context, deubiquitylation reactions may offer exciting opportunities for developing promising drug candidates due to their key roles in the proteolytic pathways as well as other biological processes [23,24]. Although the development of specific DUB inhibitors is challenging per se and still in its early stage, recently performed a series of elegant works have produced nice examples of highly selective small-molecule inhibitors for targeting USP7 and USP30 [24,39,40,41,42,43,44].

Targeting DUBs on the proteasome may also represent unique therapeutic strategies for actively regulating the proteasome-mediated proteolysis in a dynamic manner. Individual or ensemble of deubiquitination activities can exert distinct and multiple impacts on the proteasome before or throughout substrate processing (Figure 1A); such DUB-imposed regulation may render the proteasomal activities to be highly versatile, and in this sense, the proteasome acts as a critical hub as well as a rate limiting step for the ubiquitin-dependent degradation pathways [11,12]. Recent high resolution cryo-electron microscopy (cryo-EM) studies also have identified a number of conformational states of substrate-free and substrate-bound proteasomes, in which the proteasome-associated DUBs are likely to actively and differentially modulate the degradation events by sensing those conformational dynamics [4,5,12].

Among three major proteasomal DUBs, USP14 or its yeast ortholog Ubp6 is a thiol protease that is only transiently associated with the proteasome; thus, this enzyme may favor the specific conformational states of the proteasome [14,15,16,45,46,47]. Earlier genetic studies have revealed that USP14/Ubp6 is a sensitive responder to ubiquitin and proteasome stress, and also to proteotoxic stress, although in general its deficiency is tolerable for cell survival [48,49,50,51,52,53,54]. During the mouse development, however, this isopeptidase is critically involved in the motor neuron function partially through the noncatalytic mechanism [55,56,57,58]. As a therapeutic target, USP14 has been best studied in neurological disorders and cancers [23,59]. USP14 and its inhibitors (as discussed in Section 3) have been reported to regulate several pathological targets, such as Tau, ATXN3, TDP-43, GFAP, and PrP that are highly implicated in neurodegenerative diseases [16,60,61,62]. Intriguingly, apart from its inhibitory role on the proteasome, USP14 was also found to negatively regulate autophagy in mammalian cells and basal mitophagy in fly models [63,64]. USP14 expression is upregulated in several cancers including lung adenocarcinoma, ovarian cancers, esophageal squamous cell carcinoma, and pancreatic ductal adenocarcinoma, in which this enzyme is often positively correlated with tumor recurrence, metastasis, and poor patient survival [65,66,67,68,69]. Albeit seemingly a promising clinical target, the underlying mechanisms of how USP14 participates in those disease processes still remain to be elucidated.

In contrast to USP14, RPN11/PSMD14 (also known as POH1) is an essential subunit of the proteasome that belongs to JAMM/MPN metalloprotease class of DUB [19,20,70]. Recent cryo-EM studies provide the structural basis of how RPN11′s DUB activity can be coupled to substrate translocation and degradation in an ATP-dependent fashion [6,11,13,47,71,72,73]. Along with other RP components, this metalloprotease undergoes noticeable structural changes during the transitions from the substrate-free state to the substrate-processing states of the proteasome. This conformational switch drives RPN11 to be catalytically productive for the committed substrates in both repositioning on the proteasome and reshaping the local structure of its featured Ins-1 loop. Due to its strict requirement for proteasomal degradation, the genetic depletion or catalytic mutation of RPN11 causes the lethality [19,49,51,74,75,76]. Therefore, the successful targeting strategy for cancer therapy by proteasome inhibitors might be also similarly applied to RPN11-mediated inhibition of proteasomal degradation [77]. The key difference here, however, is that in contrast to the core particle (CP)-directed catalytic inhibition, RPN11 inhibition will occur on the RP, and thus is likely to show more specific effects. Besides, RPN11 has been implicated in oncogenesis as a potential drug target; its expression level is positively correlated with tumor formation and metastasis, while the genetic depletion or pharmacological inhibition showing the opposite effects–such as in hepatocellular carcinoma, multiple myeloma, breast cancer, esophageal cancer, colorectal cancer, and prostate cancer [78,79,80,81,82,83].

Like USP14, UCH37/UCH-L5 is a thiol protease class of DUB that is reversibly associated with the 19S RP of the proteasome; its binding is mediated by RPN13/ADRM1, a ubiquitin receptor which can also markedly enhance the UCH37′s activity [84,85,86]. An intriguing feature of UCH37 is that this enzyme belongs to both the proteasome and the INO80 chromatin-remodeling complex in a mutually exclusive manner; its DUB activity can be selectively activated only when bound to the proteasome [87,88,89]. UCH37 was reported to trim the distal ubiquitin from erroneously ubiquitinated proteins for their rescue [21], or it does so to release proteasome-occupying unanchored chains for the productive round of substrate loading [90]. Interestingly, a recent study demonstrated that UCH37 on the proteasome can selectively cleave the K48-linked branched chains to promote the degradation of substrates [12,22]. In any case, the exact physiological functions of UCH37 remain largely elusive. Like USP14 and RPN11, several lines of studies have reported that UCH37 expression is elevated in a number of cancers including esophageal squamous cell carcinoma, hepatocellular carcinoma, epithelial ovarian cancer, endometrial cancer, and lung adenocarcinoma, in which this protease is associated with tumor progression and poor patient survival [91,92,93,94,95,96].

## 3. Proteasomal Deubiquitinase Inhibitors

### 3.1. USP14 Inhibitors

In 2010, Finley and colleagues have identified IU1, the first highly selective inhibitor of proteasome-bound USP14 by ubiquitin-7-amido-4-methylcoumarin (Ub-AMC) hydrolysis assay-based high-throughput screening (Table 1) [16]. Because Ub-AMC is preferentially cleaved by UCH37 over USP14, the assay was performed by reconstituting ubiquitin-vinyl sulfone (Ub-VS)-pretreated human proteasome with recombinant USP14–to quench the basal UCH37 activity by Ub-VS and isolate the authentic USP14 activity on the proteasome [16,18,97]. IU1 specifically inhibits the proteasome-bound form of USP14 with an IC_50_ of 4–5 μM and shows good selectivity against a panel of eight other DUBs. A subsequent medicinal chemistry led to identification of more potent IU1 derivatives, such as IU1-2, IU1-33, and IU1-47 (IC_50_s = 1.7 μM, 1.1 μM, and 0.6 μM, respectively against USP14), all of which exhibited better selectivity over IsoT (Table 1) [62]. From this structure-activity relationship (SAR) study, some key functional moieties in the parental compound were revealed to further improve the inhibitory activity.

**Figure 2:**
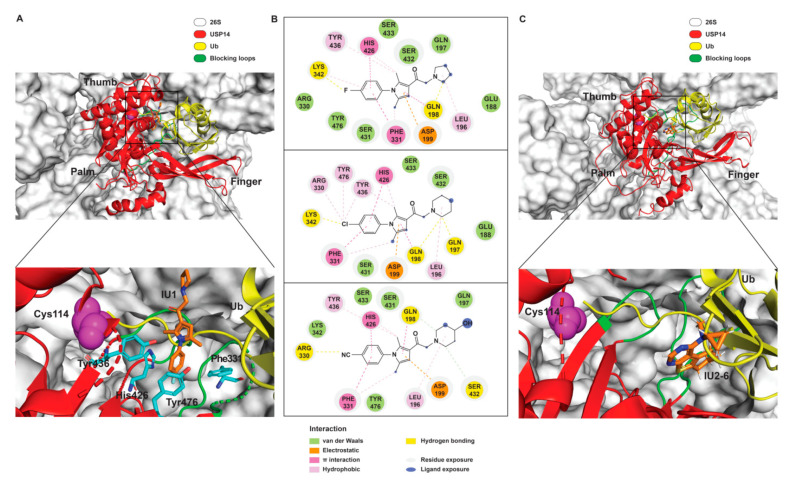
The structural illustration of USP14 in complex with its inhibitors modeled on human 26S proteasome. (**A**) The cryo-EM structure of USP14-Ubal in human 26S proteasome complex (PDB: 5GJQ) [47] was superimposed by the crystal structure of IU1-bound USP14 catalytic domain (PDB: 6IIK) [98]. IU1 is shown as an orange stick model. The catalytic Cys114 of USP14 is shown as purple spheres. The inset shows the enlarged view of the IU1 interaction with USP14. IU1 interacts with residues His426, Tyr436 and Tyr476 of USP14 (shown as cyan stick models) through van der Waals, hydrophobic, and π-π interactions. (**B**) Modeled 2D ligand interaction diagrams of IU1 analogs with USP14. The possible non-covalent interactions of IU1 with USP14 (upper panel), IU1-47 with USP14 (middle panel), and IU1-248 with USP14 (bottom panel) are drawn using BIOVIA Discovery Studio 2021 visualizer (Dassault Systèmes) [118]. Color coding of each type of non-covalent interaction is given in the key. (**C**) A modeled illustration of the interaction of IU2-6 with USP14 obtained from molecular docking studies. For docking IU2-6 on USP14, USP14 catalytic domain bound to ubiquitin aldehyde (PDB: 2AYO) [117] was selected from the Protein Data Bank (PDB), and the coordinates of ubiquitin aldehyde, heteroatoms, and water molecules were manually removed from the PDB files. The 2D structure of the IU2-6 ligand was drawn using ChemDraw software (PerkinElmer Informatics) and converted to 3D mol2 file using OpenBabel software [119]. The blind docking of IU2-6 was performed on the entire protein surface of the USP14 catalytic domain by employing the CB-dock web server (http://cao.labshare.cn/cb-dock/, accessed on 7 May 2021) [120], and generated ten docked conformations of IU2-6. Among them, one of the best-scored IU2-6 ligand docked conformation was positioned on the cryo-EM structure of USP14-Ubal in human 26S proteasome complex (PDB: 5GJQ) [47] by superimposing them. IU2-6 is shown as an orange stick model. The inset shows the enlarged view of the IU2-6 interaction with Ubal-USP14. The docked IU2-6 is positioned in between the fingers and palm regions, and also seems to interfere with the ubiquitinated substrate binding to the ubiquitin binding pocket of USP14. Color coding is as given in the key. The model figures were generated using PyMOL molecular graphics system, version 2.2.2 (Schrödinger, LLC, New York, NY, USA).

A recent study by Wang et al. reported a couple of additional IU1 analogs, IU1-206 and IU1-248, the latter having a similar IC_50_ to IU1-47 as 0.83 μM (Table 1) [98]. Notably, their co-crystal structures of USP14′s catalytic domain in complex with IU1, IU1-47, IU1-206 or IU1-248 demonstrated that IU1 series of inhibitors bind to the thumb-palm cleft pocket across the catalytic center, rather than the originally assumed catalytic site-directed inhibition (Figure 2A) [16]. This allosteric mode of inhibition hinders the entry of ubiquitin C-terminus into the catalytic center, resembling the autoinhibitory mechanism by two blocking loops (BL1 and BL2) of free USP14–which shows only sluggish activity in the absence of proteasome [16,117]. Thus, IU1 and the blocking loops are likely to compete for the similar binding sites on the apo form of USP14, and this may explain why the proteasome-unbound form of USP14 is insensitive to IU1 [11,12,16]. Recent cryo-EM structures of USP14/Ubp6-bound proteasomes revealed that their interaction may allow the ubiquitin C-terminus to be readily accessible to the catalytic cysteine by moving away the blocking loops (Figure 2A) [46,47]. 

This mode of catalytic activation can be also observed by AKT-mediated phosphorylation of the Ser432 residue on the BL2 loop of free USP14 [63]. Therefore, proteasomal activation of USP14 relieves the autoinhibitory state imposed by the blocking loops, providing the structural basis of how IU1 specifically inhibits the proteasome-bound form of USP14 [5,11,12].

The close examination of co-crystal structures reveals several non-covalent interactions between IU1 series of compounds and USP14, in which the analogs share the same binding mode [27,44,59,98]. In consistent with these studies, our own structural modeling also shows the similar binding interface between the compounds and USP14 (Figure 2A,B); (1) the π-interactions of the phenyl ring are observed with Phe331 and His426, while Tyr436 and Tyr476 make hydrophobic and van der Waals interactions with the core rings, (2) Phe331 also makes hydrophobic contact with 2-methyl group of the pyrrole ring, stabilizing the two ring structures to be perpendicular, (3) another π-stacking interaction between Asp199 and pyrrole ring is conserved, (4) the 4-Cl substitution in IU1-47 creates additional hydrophobic interactions within Phe331 pocket, thus explaining why IU1-47 is more potent than other analogs, and (5) the keto group is not engaged in making any contacts with side chains, confirming that the inhibition does not occur through the active site-directed covalent interaction. Therefore, all of these interactions fix the position of three rings in the similar configuration, but the ligand exposure at the pyrrolidine ring or 5-methyl group of the pyrrole renders these moieties to be consideration for further modification (Figure 2B).

A recent study by Palmer et al. reported a couple of IU1 analogs, 1B10 and 1D18, with better membrane permeability, and suggested a novel role of USP14 in direct MHC class I antigen presentation (Table 1) [99]. Although their exact potency was not investigated, the additional halogen substitution at the ortho-position of the phenyl ring must be tolerable in inhibitory activity, which is consistent with the previous study [62]. The optimization of IU1 series has also been actively conducted by Proteostasis Therapeutics (Cambridge, MA, USA) (now merged in Yumanity Therapeutics (Boston, MA, USA)), although the SAR studies were originally initiated from Finley and colleagues at Harvard Medical School before being licensed out to the company. They have continued to successfully develop various IU1 series of USP14 inhibitors that were published in multiple patents, as some of the examples being summarized in Table 1. Notably, a number of IU1 analogs, such as compound 162, compound 335 (SB1-B-57), compound 83, and compound 2B, showed major improvement in potency with a range of IC_50_s as <0.05~0.5 μM (Table 1) [27,100,101,102,104]. These results well corroborate with the idea that the ligand exposure sites at the pyrrole and pyrrolidine rings are amenable for compound optimization.

Another class of USP14 inhibitors, the IU2 series (IU2 was originally identified among the three strong hits from the USP14 inhibitor screening by Lee et al. in 2010 [16]), have been also actively investigated by the Finley group and Proteostasis Therapeutics (Table 1) [16,103,104,105]. IU2-6, a tricyclic thiophene pyrimidine derivative, showed 74% of USP14 inhibition at 8 μM by Ub-AMC hydrolysis assay, and further SAR studies also identified compound 3 with an IC_50_ of 0.5 μM (Table 1) [103,104,105]. Recently, Mission Therapeutics (Cambridge, UK) reported an X-ray co-crystal structure of USP14 catalytic domain bound with a thiophene pyrimidine-cored inhibitor [104]. Although the detailed structural information was not released, they proposed that the inhibitor docks at the interface between the fingers and palm regions, which is distinct from IU1 binding to the thumb-palm cleft pocket. Based on this finding, we also performed a blind docking of IU2-6 on the entire surface of the USP14 catalytic domain by using the CB-dock web server (Figure 2C) (http://cao.labshare.cn/cb-dock/, accessed on 7 May 2021) [120]. By employing publicly available USP14 structures (PDB: 2AYN, 2AYO, 5GJQ, and 6IIL), our modeling generated ten docked conformations of IU2-6 with high probability, and among the best-scored ligand docking sites, IU2-6 was positioned in between the fingers and palm domains (Figure 2C). Therefore, IU2 series of inhibitors may block the access of ubiquitinated substrates by occluding the ubiquitin binding pocket, which is consistent with the observation by Mission Therapeutics [104].

As described above, USP14 has been investigated as a potential therapeutic target to treat neurodegenerative disorders and cancers [23,59,121]. It has been well established that USP14 inhibition promotes the degradation of ubiquitinated substrates by enhancing the proteasome activity (Figure 1A) [16,62]. More importantly, IU1 or IU1-47 treatment accelerated the clearance of a variety of neuropathologic proteins, including Tau, ATXN3, TDP-43, GFAP and PrP in cells [16,60,61,62]. USP14 inhibition also confers cytoprotective effects under specific stress or neurotoxic conditions [16,122,123,124,125]. Apart from its neurological implication, emerging evidence also suggests that USP14 is highly overexpressed in several cancers, and often associated with tumor relapse and metastasis [65,66,67,68,69,126]. Accordingly, genetic downregulation or chemical inhibition of USP14 displayed anti-proliferative effects in a number of cancer models [66,67,69,126,127,128,129,130]. However, in most cases, the underlying mechanisms of USP14 inhibitor for cancer treatment remain largely elusive.

### 3.2. RPN11 Inhibitors

The first selective RPN11 inhibitor was reported by the Deshaies and Cohen groups in 2017 [106,107]. To measure robust RPN11 activity, the researchers established an elegant fluorescence polarization-based assay with the tandem tetraubiquitin tagged-peptide Oregon Green (Ub_4_-pepOG) as the DUB substrate. By employing two types of chemical libraries–1) metal binding pharmacophores-focused fragment library of 351 compounds and 2) high-throughput screening library of 330,000 compounds, they identified the hits of 8-thioquinoline (8TQ) and H18, which is actually a thioester derivative of 8TQ (Table 1). They also demonstrated that 8TQ and H18 inhibit RPN11 by chelating the metal coordination of the active site Zn^2+^ ion [106,107]. Subsequent SAR study was conducted to optimize the functional moiety of 8TQ, and the lead compound capzimin was successfully developed (Table 1). Capzimin (8-mercapto-N-(2-(thiazol-2-yl)ethyl)quinoline-3-carboxamide) was seven-fold more potent than 8TQ for RPN11 (IC_50_ = 0.34 μM), and showed good selectivity over other JAMM metalloproteases with a range of 6 to 80-fold in IC_50_s. Intriguingly, the inhibitory mechanism of capzimin is reversible and uncompetitive for RPN11, while displaying the competitive inhibition against AMSH and BRCC36. When treated in cells, capzimin strongly elicited the formation of aggresomes and the accumulation of ubiquitinated conjugates, UbG76V-GFP model substrate, and endogenous proteasomal substrates, as observed in proteasome inhibitor treatment.

Additional RPN11 inhibitors were also reported recently right after capzimin. Lauinger et al. revealed thiolutin, a bicyclic antibiotic dithiolopyrrolone compound as a general JAMM metalloprotease inhibitor (Table 1) [108]. Thiolutin was originally studied for its inhibitory activity against bacterial or fungal RNA polymerases, but surprisingly the researchers found that this compound can inhibit RPN11 and other JAMM metalloproteases such as CSN5, AMSH, and BRCC36 (IC_50_s = 0.53 μM, 6.16 μM, 3.96 μM and 0.79 μM, respectively), and those inhibitory activities are apparently coming from the catalytic Zn^2+^ ion chelation. In 2018, Deshaies and colleagues reported another type of RPN11 inhibitor, the epidithiodiketopiperazine (ETP) compounds (Table 1) [109]. ETPs are the virulence factors as the second metabolites that are generated from Aspergillus fumigatus, and among them, gliotoxin is known as the most potent toxin. By developing a protein breakdown assay with fluorescent Ub_n_GST-Wbp2 as the model substrate, the researchers found that ETPs can block proteasome function by targeting the RPN11 DUB activity. Like capzimin and thiolutin, the ETP compounds inhibit RPN11 by chelating the active site Zn^2+^. Among the tested ETPs, SOP6 is the core scaffold compound that retains the low micromolar IC_50_s of potency against JAMM domain proteases (IC_50_s = 3.8 μM to RPN11, 2.9 μM to CSN5, and 2.1 μM to AMSH, respectively), and SOP11 shows slightly higher potency than SOP6 (IC_50_s = 1.3 μM to RPN11, 0.6 μM to CSN5, and 0.9 μM to AMSH, respectively) (Table 1). Importantly, SOP11 was considered to be the most promising candidate because when treated to the cells, this inhibitor did not noticeably inhibit CSN5 activity, while closely mimicking capzimin in triggering a strong unfolded protein response and inducing the accumulation of ubiquitin conjugates.

**Figure 3:**
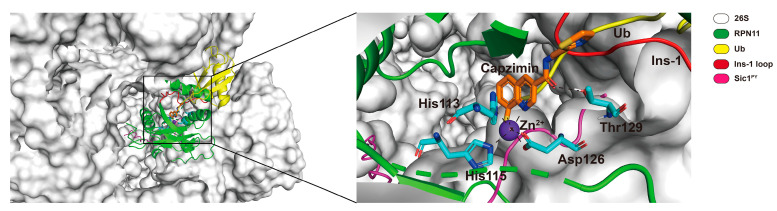
An illustration of RPN11-capzimin docked complex structure modeled on the human 26S proteasome. The RPN11-capzimin docked complex [131] was positioned on the cryo-EM structure of USP14-Ubal in human 26S proteasome complex (PDB: 5GJQ) by superimposing each other. Similarly, polyubiquitylated Sic^PY^ (PDB: 6MSE) [73] was positioned on the modeled structure. Capzimin is shown as an orange stick model. The catalytic His113 and His115 residues of RPN11 are shown as cyan stick models. Zn^2+^ ion is shown as a purple sphere. The inset shows the enlarged view of the RPN11-capzimin complex. Capzimin interacts with Thr129 of RPN11 (shown as a cyan stick model) through hydrogen bond, and also makes a bidentate interaction with Zn^2+^. The position of capzimin may block the access of isopeptide linkage of the ubiquitin conjugates to the catalytic zinc-binding site or interact with the residues in the ubiquitin binding pocket. Color coding is as given in the key. The model figures were generated using PyMOL molecular graphics system, version 2.2.2 (Schrödinger, LLC, New York, NY, USA). Some residues or regions were removed to show clear interaction between RPN11 and capzimin.

So far, there is no available RPN11 structure in complex with its inhibitor resolved by X-ray crystallography, NMR, or cryo-EM. However, a recent study reported the computational modeling of interaction between RPN11 and capzimin by molecular dynamics simulation [131]. We received the modeling information of RPN11-capzimin from the researchers (kindly provided by V. Kumar and M. Stein, Max Planck Institute, Magdeburg, Germany), and performed further analysis as following. The docked conformation from the modeling was positioned on the cryo-EM structure of USP14-Ubal-human 26S proteasome complex (PDB: 5GJQ) by superimposing the modeled RPN11-capzimin complex with proteasome-associated RPN11 found in structure (Figure 3) [47]. Also, ubiquitylated Sic1^PY^ substrate (PDB: 6MSE) was similarly placed on the USP14-Ubal-proteasome cryo-EM structure [73]. From this modeling, we confirmed that capzimin makes a bidentate coordination with the active site Zn^2+^ and also forms a stable hydrogen bonding with the side chain of Thr129, as observed by Kumar et al. In addition to chelating Zn^2+^ ion, capzimin at the docked position may interact with the residues in the ubiquitin binding site or block the access of isopeptide linkage of ubiquitinated substrate to the catalytic site (Figure 3).

Due to its strict degradation-coupled activity, RPN11 inhibition may exert effects comparable to the CP-targeting proteasome inhibitors for cancer therapy. However, distinct from canonical proteasome inhibition, RPN11-mediated inhibition would occur at RP and thus may provide alternative opportunities to treat cancers, especially the ones refractory to CP-proteolysis inhibitors. Like proteasome inhibitors such as bortezomib or carfilzomib, RPN11 inhibitors strongly suppress proteasomal degradation of substrates in vitro and in cells [106,108,109]. Importantly, capzimin treatment showed similar growth inhibition of bortezomib-resistant retinal pigment epithelial cells compared to their original counterpart. By screening against the 60 NCI panel of cancer cell lines, capzimin exhibited the potent growth inhibition of leukemia cells (SR and K562) as well as solid tumors (NCI-4460 and MCF7) with subsequent induction of apoptosis [106]. SOP11 also induced apoptosis of HCT116 human colon cancer cells, and showed the similar growth inhibition against original and bortezomib-resistant retinal pigment epithelial cells [109]. Besides, a number of recent findings indicate that genetic depletion or pharmacological inhibition of RPN11 strongly antagonizes the growth of several types of cancers or tumor cell invasion, validating the RPN11 metalloprotease as a promising drug target for cancer therapy [80,81,132,133].

### 3.3. Other Proteasomal Deubiquitinase Inhibitors

UCH37 specific inhibitors have not been developed yet, and so in this Section, we will primarily discuss some nonspecific DUB inhibitors which also target UCH37 activity. b-AP15 (originally known as NSC687852) was first identified from cell-based chemical screening by the Linder group as a small-molecule that can induce lysosomal and p53-independent apoptosis (Table 1) [134,135]. In 2011, the same group reported that b-AP15 inhibits 19S RP DUB activity, specifically USP14 and UCH37, and such dual inhibition leads to the accumulation of polyubiquitinated conjugates through proteasome inhibition [110]. The reported IC_50_s of b-AP15 against 19S RP have been somewhat inconsistent in the literatures: 2.1 μM or 16.8 μM for Ub-AMC [110,111,136], or 6.5 μM for ubiquitin-rhodamine (Ub-Rho) [111]. The researchers also developed VLX1570, an azepane-cored b-AP derivative by performing SAR studies (Table 1) [111]. IC_50_s of VLX1570 against 19S RP DUB activity were obtained as 13 μM for Ub-AMC and 6.4 μM for Ub-Rho [111]. The authors argued that VLX1570 may inhibit those two thiol protease of DUBs by making Michael’s addition-based covalent interactions with the catalytic cysteines despite its reversible binding mode [136,137]. Curiously, they further found that VLX1570 preferentially inhibits USP14 over UCH37 in active site-directed ubiquitin probe competition assays, which is distinct from its comparable inhibition of both of the DUBs from the fluorescent ubiquitin adduct cleavage assays [137].

WP1130 (also known as degrasyn) was first developed by the Donato group from cell-based chemical screening to seek for superior Janus Kinase 2 (Jak2) inhibitors, and indeed this compound is a structural analog of AG490, one of the most well-known Jak2 inhibitors (Table 1) [138,139]. WP1130 strongly suppressed Jak2-dependent cytokine signaling at 50 to 100-fold less concentration than AG490 but surprisingly did so without direct inhibition of Jak2 activity [139]. By employing activity-based ubiquitin probe with cell lysates, the same group reported that WP1130 inhibits a panel of DUB activities, such as USP9x, USP5, USP14, and UCH37 with a range of IC_50_s at <5 to 10 μM [112]. WP1130-mediated suppression of Jak2/STAT3 signaling might be at least partially explained by Jak2-specific DUB inhibition as observed by induction of K63-linked Jak2 ubiquitination after the compound treatment [139]. On the other hand, when Ritorto et al. recently performed MALDI-TOF mass spectrometry analysis for profiling 42 DUBs with activity-based diubiquitin probes, the researchers found that WP1130 displays highly promiscuous inhibition of assorted DUBs that were not identified previously, and in which over 10 DUBs turned out to be more sensitive than USP9x to WP1130 treatment [140]. AC17, a 4-arylidene curcumin analog, was also reported to be another 19S RP DUB inhibitor by a Chinese group (Table 1) [113]. AC17 was known to inhibit IκB kinase through unknown mechanisms, but in their study, the authors found that similar to b-AP15, the compound serves as an atypical proteasome inhibitor by blocking the 19S RP DUB activity with an IC_50_ of 4.23 μM for Ub-AMC. However, the potency and selectivity of AC17 against individual proteasomal or non-proteasomal DUBs remain to be determined. Lastly, Liu et al. reported that auranofin, an FDA-approved gold-containing drug that is clinically used to treat rheumatoid arthritis, can exert proteasome inhibitory effect by inhibiting proteasome-associated DUBs, USP14 and UCH37 (Table 1) [114]. By performing Ub-AMC hydrolysis and Ub-VS labeling assays, the researchers observed that auranofin significantly inhibits both USP14 and UCH37 approximately at tens of μM concentration. However, in 2019, the Linder group argued that DUB inhibition by auranofin may not be a primary cause of its cytotoxicity towards cancer cells [116]. Instead, they demonstrated that at pharmacological doses of auranofin, selenoprotein thioredoxin reductase (TrxR) is the main target, while proteasomal DUB inhibition is rather off-target effect, pointing to an important specificity issue when applying promiscuous proteasomal DUB inhibitors [115,116].

As noted above, UCH37 may serve as a therapeutic target because this DUB is upregulated in a number of cancer models including ovarian cancer and hepatocellular carcinoma, which is often associated with tumor progression and invasion [92,93]. Despite the lack of selective UCH37 inhibitor, the developed multi-targeting DUB inhibitors (which also targeting UCH37) may still provide certain clinical benefits for cancer therapy. In fact, b-AP15 and VLX1570 disturb cellular proliferation and induce apoptosis in several types of solid tumors, leukemia, bortezomib-resistant cancer cells, and also mouse tumor xenograft [110,137,141,142,143,144,145]. Especially, VLX1570 was tested for phase I clinical trials to treat refractory multiple myeloma patients (currently suspended due to high toxicity) [146]. Likewise, WP1130 exerted strong anti-proliferative effects and apoptotic induction towards several cancer cells including mantle cell lymphoma, chronic myelogenous leukemia, and T-cell acute lymphoblastic leukemia [112,147,148]. AC17 also showed the suppression of tumor growth in a murine xenograft model of human lung cancer A549 by p53 reactivation and NF-κB blockage [113], and auranofin treatment was cytotoxic to HepG2 and MCF-7 cancer cells by inhibiting proteasome function, although its on target effect on proteasome for cytotoxicity should be further addressed [114,116]. In any cases, however, the exact contribution of UCH37 inhibition remain to be validated for applying these nonspecific proteasomal DUB inhibitors to the disease models.

## 4. Concluding Remarks

Here we summarize the up-to-date progress in developing proteasomal DUB targeting inhibitors and their therapeutic potential for applying to human pathophysiology. Active regulation of proteasome function by DUB inhibition may provide unprecedented and unique proteolysis-based therapeutic opportunities to treat a number of diseases that have been intractable by conventional targeting strategies. Despite the initial skepticism, the clinical success of proteasome inhibitors in cancer treatment highlights the value of the UPS system as an important drug target. Therefore, specific DUB inhibition on the proteasome should be reasonable and certainly beneficial in a way that each proteasome-associated DUB may serve as a more specific therapeutic target in the context of different disease conditions. In fact, USP14, RPN11, and UCH37 on the proteasome are each dynamically engaged in discrete substrate processing steps by sensing the diverse conformational states of the proteasomes, and by doing that, they often influence the proteolytic outcome differently [11,12]. In this sense, DUBs on the proteasome may act as checkpoints or gatekeepers for ubiquitin-mediated degradation pathways, and in here, DUB inhibition is most likely capable of overriding the DUB-imposed restriction toward acquiring actively controlled and tailored proteolytic outputs. USP14 inhibitors, for example, have been attempted to treat a variety of disease models including neurodegenerative diseases, cancers, and others [149]. USP14 inhibition promotes the clearance of neuropathic substrates by increasing proteasome activity, which would be among the underlying mechanisms for treating the neurological disorders. By contrast, it remains to be seen whether anti-cancer effects by USP14 inhibitors can be also mediated, at least in part by enhanced proteasomal degradation. RPN11 inhibitors, on the other hand, will stall the proteasome substrates upstream of CP and antagonize their degradation. Therefore, RPN11 inhibition will offer an alternative way to treat cancers that are resistant to CP-targeting proteasome inhibitors. Specific UCH37 inhibitors have yet to be developed, but this protease also seems to be promising as a drug target, such as in cancer therapy. In the coming decade, we might witness the development of the first-in-class drug of targeting proteasomal DUBs to treat human diseases. In any cases, however, the endeavor to develop small-molecule inhibitors targeting DUBs on the proteasome will greatly improve our understanding in deubiquitination biology.

## Figures and Tables

**Table 1:** Representative examples of the reported proteasomal deubiquitinase inhibitors.

Target	Compound Name	Structure	Notes	Reference
USP14	IU1	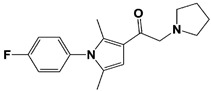	IC_50_: 4.7 μM (Ub-AMC)	Lee et al., 2010 [16]
	IU1-2	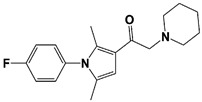	IC_50_: 1.7 μM (Ub-AMC)	Boselli et al., 2017 [62]
	IU1-33	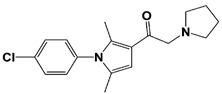	IC_50_: 1.1 μM (Ub-AMC)	
	IU1-47	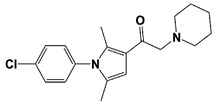	IC_50_: 0.6 μM (Ub-AMC)	
	IU1-206	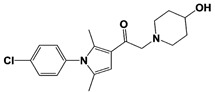	N/A	Wang et al., 2018 [98]
	IU1-248	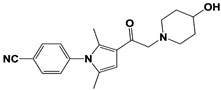	IC_50_: 0.83 μM (Ub-AMC)	
	1B10	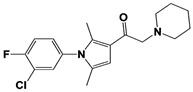	N/A	Palmer et al., 2018 [99]
	1D18	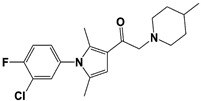	N/A	
	Compound 162	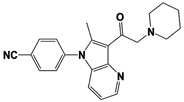	IC_50_: <0.5 μM (Ub-AMC)	WO/2015/073528 [100]
	Compound 335 (SB1-B-57)	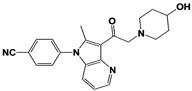	IC_50_: <0.5 μM (Ub-AMC)	
	Compound 83	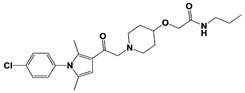	IC_50_: <0.5 μM (Ub-AMC)	WO/2020/006269 [101]
	Compound 2B	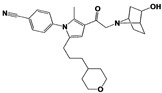	IC_50_: <0.05 μM (Ub-AMC)	WO/2020/006296 [102]
	IU2-6	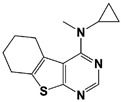	74% inhibition at 8 μM (Ub-AMC)	WO/2012/012712 [103]; Kemp, 2016 [104]
	Compound 3	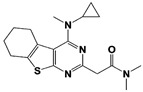	IC_50_: 0.5 μM (Ub-AMC)	WO/2013/112651 [105]; Kemp, 2016 [104]
RPN11	8-TQ *	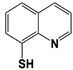	IC_50_: 2.4 μM (Ub_4_-pepOG)	Li et al., 2017 [106]; Perez et al., 2017 [107]
	Capzimin	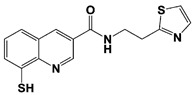	IC_50_: 0.34 μM (Ub_4_-pepOG)	
	Thiolutin *	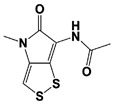	IC_50_: 0.53 μM (Ub_4_-pepOG)	Lauinger et al., 2017 [108]
	SOP6 *	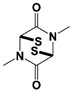	IC_50_: 3.8 μM (Fluorescent Ub_n_GST-Wbp2)	Li et al., 2018 [109]
	SOP11 *	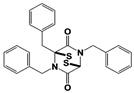	IC_50_: 1.3 μM (Fluorescent Ub_n_GST-Wbp2)	
Promiscuous proteasomal DUB inhibitors	b-AP15 (USP14/ UCH37)	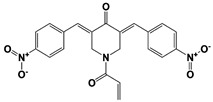	19S RP IC_50_: 6.5 μM (Ub-Rho)	D‘Arcy et al., 2011 [110]; Wang et al., 2015 [111]
	VLX1570 (USP14/ UCH37)	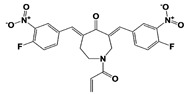	19S RP IC_50_: 6.4 μM (Ub-Rho)	Wang et al., 2015 [111]
	WP1130 (USP9x/USP5/ USP14/UCH37)	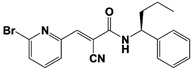	IC_50_s: <5~10 μM (Ub-AMC & Ub-VS)	Kapuria et al., 2010 [112]
	AC17 (19S RP)	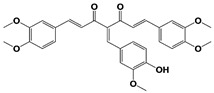	19S RP IC_50_: 4.23 μM (Ub-AMC)	Zhou et al., 2013 [113]
	Auranofin ** (TrxR/19S RP; 19S RP at higher dosage than TrxR)	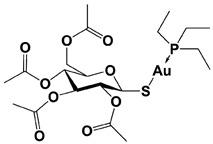	TrxR system inhibition at ~1 μM (HCT-116 cell) Reduced 19S RP labeling at 5 μM (Ub-VS)	Liu et al., 2014 [114]; Stafford et al., 2018 [115]; Zhang et al., 2019 [116]

* These inhibitors also inhibit other JAMM metalloproteases. ** This inhibitor may have its non-DUB target in pharmacological dosage.

## Data Availability

Not applicable.

## Abbreviations

UPS	Ubiquitin-proteasome system
DUB	Deubiquitinase
RP	Regulatory particle
CP	Core particle
PROTAC	Proteolysis targeting chimera
Cryo-EM	Cryo-electron microscopy
Ub-AMC	Ubiquitin-7-amido-4-methylcoumarin
Ub-Rho	Ubiquitin-rhodamine
Ub-VS	Ubiquitin-vinyl sulfone
Jak2	Janus kinase 2
TrxR	Thioredoxin reductase
